# Improving Cooling Performance of Injection Molding Tool with Conformal Cooling Channel by Adding Hybrid Fillers

**DOI:** 10.3390/polym13081224

**Published:** 2021-04-10

**Authors:** Chil-Chyuan Kuo, Wei-Hua Chen

**Affiliations:** 1 Department of Mechanical Engineering, Ming Chi University of Technology, New Taipei City 243, Taiwan; M03118013@mail.mcut.edu.tw; 2 Research Center for Intelligent Medical Devices, Ming Chi University of Technology, No. 84, Gungjuan Road, New Taipei City 243, Taiwan

**Keywords:** silicone rubber mold, thermal conductivity, filler, cooling time, relative error rate

## Abstract

Silicone rubber mold (SRM) is capable of reducing the cost and time in a new product development phase and has many applications for the pilot runs. Unfortunately, the SRM after injection molding has a poor cooling efficiency due to its low thermal conductivity. To improve the cooling efficiency, the thermal conductivity of the SRM was improved by adding fillers into the SRM. An optimal recipe for fabricating a high cooling efficiency low-pressure injection mold with conformal cooling channel fabricated by fused deposition modeling technology was proposed and implemented. This study proposes a recipe combining 52.6 wt.% aluminum powder, 5.3 wt.% graphite powder, and 42.1 wt.% liquid silicon rubber can be used to make SRM with excellent cooling efficiency. The price–performance ratio of this SRM made by the proposed recipe is around 55. The thermal conductivity of the SRM made by the proposed recipe can be increased by up to 77.6% compared with convention SRM. In addition, the actual cooling time of the injection molded product can be shortened up to 69.1% compared with the conventional SRM. The actual cooling time obtained by the experiment is in good agreement with the simulation results with the relative error rate about 20%.

## 1. Introduction

The digital manufacturing technology (DMT) plays an important role in the precision machinery. Additive manufacturing (AM) [1,2,3,4] is one of DMTs with some advantages, such as variety, complexity, flexibility, reducing energy usage, less materials waste, little lead time compared with conventional manufacturing processes. AM technology can provide the mold or die designers to verify a design in a matter of hours. Rapid tooling (RT) technology [5,6,7,8] is divided into soft or hard tooling. Tooling for low volume manufacturing runs is known as soft tooling (ST). The tools in ST are made from materials, such as silicone rubber or epoxy resin. According to the practical experience, the ST is easier to perform compared with conventional tooling steels. Among the ST technology, the silicone rubber mold (SRM) was often used to fabricate low-pressure wax injection molds (LPWIM) for manufacturing wax patterns with complex geometrical shapes since low-pressure wax injection molding is one of the common manufacturing processes for producing wax patterns [9,10,11]. Yuan et al. [12] investigated the rheological behavior of silicone rubber (SR) used in sheet metal flexible-die forming. It was found that the numerical analysis results of viscous pressure stretching are in good agreement with the experimental results. In addition, the higher viscous pressure can improve sheet formability. Wu et al. [13] investigated the macro–micro contact properties of SR by experiment and the finite element method. A new tester of macro contact properties of rubber seals was developed for measuring the contact area and contact force in real time. It was found that the simulation results are in good agreement with the experimental result. He et al. [14] developed a novel arc-spraying robot for rapid tooling. This robot can carry out the tooling process automatically and efficiently fully based on the sliced data of the master pattern. Rajaguru et al. [15] developed a rapid tooling for low-volume production of plastic parts using rapid prototyping technology and electroless nickel plating. The service life of the developed rapid tooling is longer under normal plastic processing conditions. Issa et al. [16] developed sensor elements for passively compliant gripper using conductive silicone rubber. It was found that the gripper can accommodate to any irregular and sensitive grasping object and carry light object. Ou et al. [17] investigated the filling and curing phases of bi-injection molding of liquid silicone rubbers.

It was found that the simulation results fit the shear viscosity behaviors of silicone LSR4350 well and the proposed Isayev-Deng model fits the curing kinetic reaction of elastomer materials well. Ou et al. [18] developed mathematical models for the thermo-kinetic parameters of silicone elastomers.

It was found that the simulation results predicted the mold filling and cure degree state into the die mold cavity accurately. Yang et al. [19] developed a microfabrication process to replicate high-aspect-ratio microstructures using silicone rubber micro molds. It was found that aspect ratio of 15:1 for a single piece and larger aspect ratio can be achieved. The proposed replication process provides low cost, mass production of high-aspect-ratio microstructures. Thian et al. [20] fabricated microfluidic channel using silicone rubber with vacuum casting. It was found that the casting of microfluidic channel via vacuum casting provides high repeatability. Glocke and Wiseman [21] fabricated micro-Fresnel zone plates using silicone rubber. A high quality 10-μm-thick zone plates having 100 zones and a 5-μm-wide outer zone was fabricated. Christian et al. [22] fabricated a jawbone mold using silicone rubber. This study highlighted the importance of minimizing reactant viscosity in producing satisfactory polymer-glass bond. Ching et al. [23] fabricated a facial prosthetic model using silicone rubber. It was found that aesthetically matching and comfort of the prosthesis play a leading role to the success of prosthetic implants. This paper also presented a novel approach for making a prosthetic ear model. Maji et al. [24] developed a patient-specific craniofacial implant using rapid tooling technology. It was found that results of this study could assist a surgeon in preplanning an actual operation. Kozior et al. [25] presented the results of tests of surface waviness of samples made in the powder bed fusion technology with stainless steel powder. Results showed that the orientation of model arrangement has an impact on the quality of the technological surface texture. Kozior and Kundera [26] presented the research results of the surface texture measurement of models built by the fused deposition modeling (FDM) technology with ABS plastic. It was found that the direction of placing the models on the building platform affects the value of two-dimensional and spatial parameters of the geometric surface texture.

Generally, the injection molding involves filling, packing, cooling, and ejection four major stages. Especially, the cooling stage is the most dominant step of the cycle time. It is well known that the conformal cooling channel (CCC) provides better cooling performances than the conventional cooling channel. The productivity can be enhanced by the LPWIM with CCC [27] because the cooling can be reduced significantly during the cooling stage. Kanbur al. [28] reviewed the design and optimization of CCC for injection molding. This study also discussed the 3D models of CCC and relevant mold materials. Vojnova [29] introduced the benefits of molds with conformal cooling systems in the injection molding process. This study developed technological parameters on the quality of the injection molded parts by comparing the length of the production cycle based on cooling duration or individual types of cooling systems. Kitayama et al. [30] examined the cooling efficiency of conformal cooling channel in plastic injection molding (PIM) numerically and experimentally. It was found that the structural optimization is an important approach to determine the CCC. Holker and Tekkaya [31] developed extrusion dies with conformal cooling channels for increasing the productivity in the hot aluminum (Al) extrusion. It was found that the hybrid tools withstand the high mechanical and thermal loads which occur during hot aluminum extrusion. Lim et al. [32] proposed a method for designing the cooling channel by means of the energy balance principle and arrangement method. It was found that average tensile strengths of roof side products manufactured with the cooling times of 12 and 9 s were 1487 and 1488 MPa, respectively. Wang et al. [33] employed optimization of mold with spherical spiral conformal cooling system and product structure to reduce service stress of the injection molded products. This study indicated that geometrical structure should be designed according to the assembly and service conditions. In addition, molding defects, such as warpage, residual stress need to be considered in mechanical analysis. Brooks and Brigden proposed a concept for designing the conformal cooling layers with self-supporting lattices [34]. A case study of the injection molding of a plastic enclosure was used to compare the performance of conformal cooling layers with conventional cooling channels and conformal cooling channels. It was found that the conformal layers reduce the cooling time by 26.34% over conventional cooling channels.

However, the cooling efficiency of the mold made of SR was limited to the thermal conductivity of the materials. Therefore, enhancing the cooling efficiency of the SRM with CCC is an important research issue. In this study, a recipe for fabricating high cooling efficiency SRM was investigated. The five different fillers were added into the SRM to investigate the changes in thermal conductivity. The optimal recipe for fabricating SRM with high thermal conductivity was investigated experimentally and employed to fabricate high cooling efficiency SRM. In this study, the computer-aided engineering (CAE) simulation software named Moldex 3D was used since the design process of CCCs is difficult than the conventional cooling channels. The simulation software was used to predict cooling time of the injection molded product, part temperature distribution, mold temperature distribution, and warpage of the wax patterns. To validate the performance of the high cooling efficiency SRM, LPWIM were carried out for batch production of wax patterns.

## 2. Experimental Details

**Figure 1:**
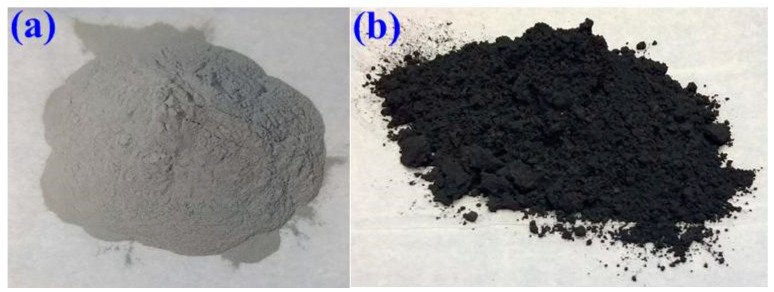
Photos of the (**a**) aluminum (Al) and (**b**) graphite (G) powders.

**Figure 2:**
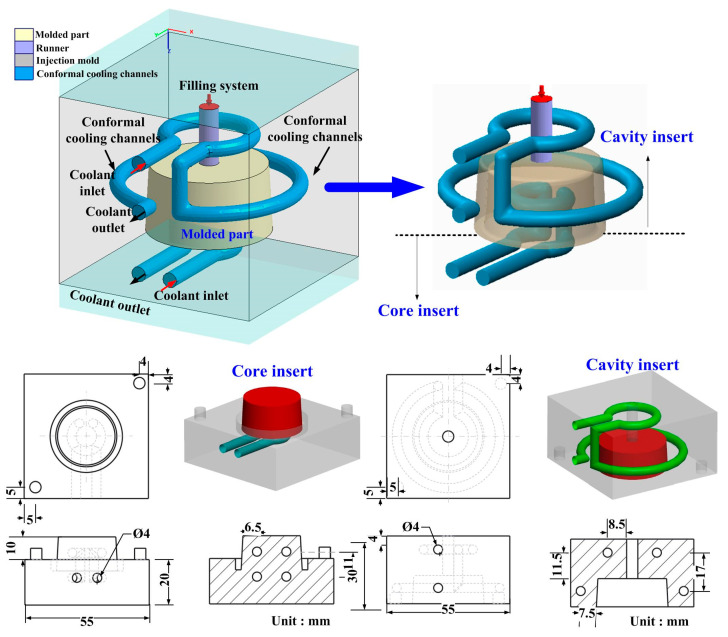
Relationship of filling system, injection molded product and conformal cooling channel (CCC) of both core and cavity plates.

**Figure 3:**
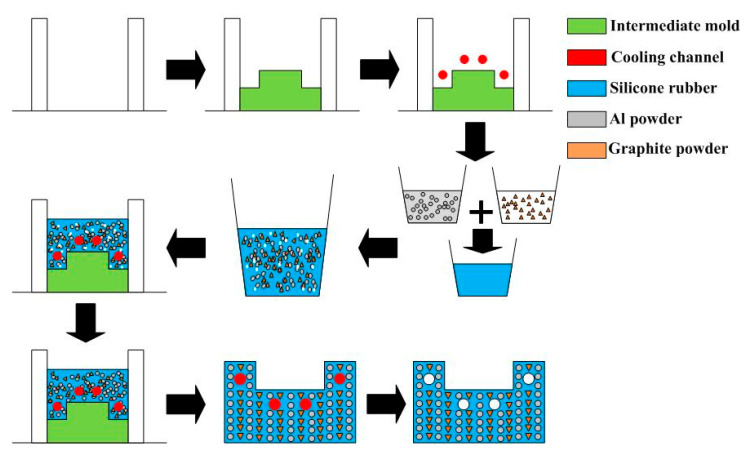
Schematic illustrations of the production process of a high cooling efficiency low-pressure injection mold with CCC.

In this study, the product, SRM, and CCC were designed using SolidWorks software (SolidWizard Technology Inc, Taipei City, Taiwan). The injection molded product is a pipe end cap with dimensions of 32 mm in the upper outer diameter, 27 mm in the lower outer diameter, 17.5 mm in height, and 2.5 mm in wall thickness. The main reason for choosing the pipe end cap as an injection molded product is that the top edge of the water cup is set as a parting surface, which can easily disassemble the core and cavity inserts. Moreover, the CCC geometries designed for core and cavity insert do not overlap. Thus, choosing the pipe end cap as an injection molded product can demonstrate the substantial benefits of rapid injection tool with CCC. The process parameters for the simulation include filling time of 2.02 s, injection pressure of 0.06 MPa, mold temperature of 25 °C, wax melting temperature of 99 °C, coolant temperatures of 25 °C, and an ejection temperature of 30 °C. To improve the thermal property of the SRM must be improved since it is a key factor on the cooling characteristics. In this study, five different fillers, namely aluminum (Al) powder, copper (Cu) powder and graphite (G) powder, iron (Fe) powder, and carbon black (CB) powder were added into the liquid silicone rubber to investigate the effects of different fillers on the thermal conductivity. Figure 1 shows the photos of Al and G powders. The average particle sizes of five different fillers are 48 µm, 75 µm, 13 µm, 48 µm, and 28 µm, respectively. The material costs of Al, Cu, G, Fe, and CB powders are NTD 0.5/g, 0.19/g, 0.9/g, 0.8/g, and 0.53/g, respectively. The CAE software named Moldex 3D (R16 SP3OR, CoreTech System Inc., New Taipei City, Taiwan) was employed to predict the cooling time of injection molded products, the mold temperature difference, the part temperature difference, and warpage of injection molded products. The standard process parameters for the simulation include injection pressure of 0.06 MPa, wax melting temperature of 99 °C, coolant flow rate of 130 cc/s, inlet temperatures of 25 °C, and an ejection temperature of 30 °C. In this study, the boundary layer mesh was used to ensure the accuracy of simulation results because it is suitable for simulation models with intricate geometries. Based on the design guideline of CCC [35], the cooling channel diameter, the distance between the wall of cooling channel to the mold surface, and the pitch distance between central lines of cooling channels are 4 mm, 7.5 mm, and 17 mm, respectively. Figure 2 shows the relationship of filling system, injection molded product and CCC of both core and cavity plates. In this study, the polyvinyl butyral (PVB) resin was selected as the material of CCC since it can be removed by methyl alcohol. The FDM machine (SAFEWAY 3D, New Taipei City, Taiwan) was used to fabricate the CCC because it provides the flexibility in the fabrication of complex CCC compared with conventional machining technologies. The process parameters for making the CCC are printing temperature of 190–210 °C, the hot bed temperature of 60 °C, the printing speed of 20 mm/s, and the layer thickness of 0.2 mm. Figure 3 shows the schematic illustrations of the production process of a high cooling efficiency low-pressure injection mold with CCC. The silicone rubber (KE-1310ST, Shin Etsu Inc., Tokyo, Japan) and curing agent (CAT-1310S, Shin Etsu Inc., Tokyo, Japan) were mixed in a weight ratio of 10:1 to fabricate an SRM. A vacuum machine (F-600, Feiling, Inc., Taoyuan, Taiwan) was used to extract the air-bubbles resulting from the mixing process under vacuum conditions.

**Figure 4:**
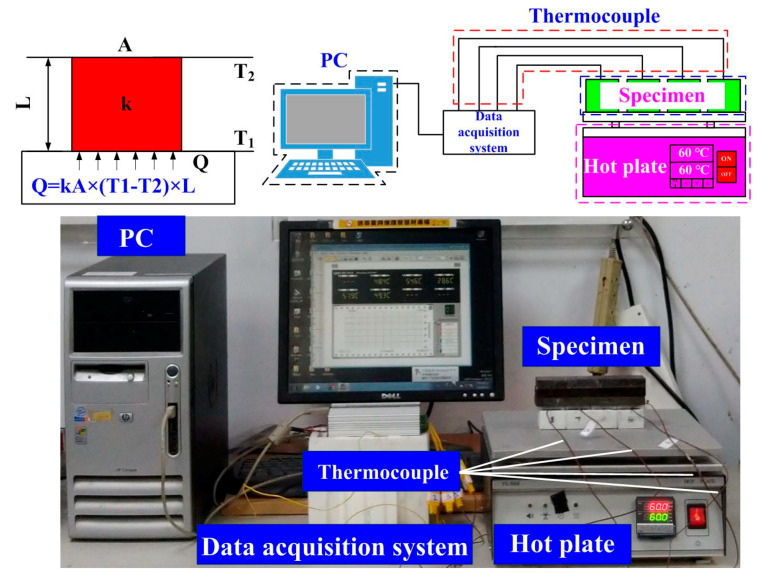
Experimental setup for measuring thermal conductivity of the silicone rubber with different fillers.

Phase identification was carried out by X-ray diffraction (XRD) (D8 ADVANCE, Bruker Inc., New Taipei City, Taiwan). X-raying was performed in the geometry of a parallel beam with Cu Kα radiation. In addition, the chemical composition of the fabricated SR molds was examined using an energy-dispersive X-ray spectroscopy (EDS). To investigate the thermal conductivity of the silicone rubber with different fillers, the thermal conductivity was carried out. The length, width and height of the test specimen are 25 × 25 × 25 mm^3^, respectively. The test specimens were placed on the top of the hot plate (YS-300S, YOTEC Inc., New Taipei City, Taiwan) and heated at 60 °C. The K-type thermocouple was placed on the top of the test specimens to record the temperature history using a data acquisition system (MRD-8002L, IDEA System Inc., Taoyuan, Taiwan). Figure 4 shows the experimental setup for measuring thermal conductivity of the silicone rubber with different fillers. In this study, the wax (K512, Kato Inc., Taoyuan, Taiwan) was used as molding materials. To investigate the cooling efficiency of the fabricated SRM with different fillers, the low-pressure wax injection molding was performed using a low-pressure wax injection machine (LPWIM) (0660, W&W Inc., Taoyuan, Taiwan). To validate the performance of the high cooling performance SRM with CCC, an experimental setup was developed and implemented. This system was composed of three k-type thermocouples (C071009-079, Cheng Tay Inc., New Taipei City, Taiwan), a water reservoir with a thermo-electric cooler (TEC12706AJ, Caijia Inc., New Taipei City, Taiwan), a temperature controller (JCM-33A, Shinko Inc., New Taipei City, Taiwan), and a data acquisition system. The water was used as cooling medium in this study. The inlet coolant temperature was kept at room temperature. Temperature histories of the molded wax patterns were recorded by a data acquisition system. The ejection temperature of the molded wax patterns was set at 30 °C via a series of test runs. The cooling time can be determined according to the temperature histories of the molded wax patterns after LPWIM.

## 3. Results and Discussion

**Figure 5:**
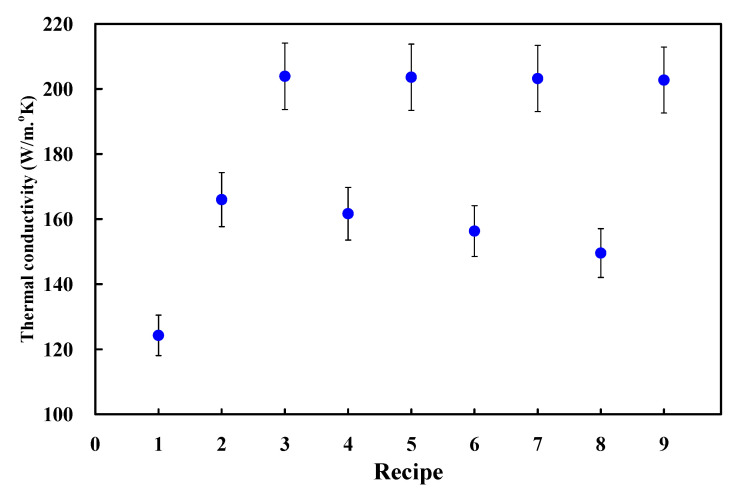
Recipe as a function of thermal conductivity.

According to preliminary experiments [36], the thermal conductivity of pure silicone rubber is approximately 21.4 W/m-K. The thermal conductivities of the silicone rubber with 60 wt.% Al powder, 80 wt.% Fe powder, 70 wt.% Cu powder, 20 wt.% G powder, and 15 wt.% CB powder are approximately 29.4 W/m-K, 41.3 W/m-K, 36.9 W/m-K, 33.0 W/m-K, and 23.4 W/m-K, respectively. Three phenomena were found: (a) the thermal conductivity of the SRM was improved significantly when 80 wt.% Fe powder was added into SR. However, the SRM affected by the coolant is prone to rust when the Fe powder was selected as filler. This drawback of the SRM will affect the cooling performance in the LPWIM; (b) the thermal conductivity of the SRM was not improved greatly when the CB powder was selected as filler. Improvement in thermal conductivity of the SRM is only about 9.3%; (c) the thermal conductivity of the SRM was improved to 36.9 W/m-K when the Cu powder was selected as filler. However, the cost of Cu powder is expensive compared with other powders. Based on the above analysis, Al and G powders were selected as filler to develop high cooling efficiency SRM with CCC. Table 1 shows the planning table for silicon rubber adding different weight ratios of Al powder and G powder. Figure 5 shows the recipe as a function of thermal conductivity. According to the thermal conductivity, the SRM made with the recipes 3, 5, 7, and 9 is the first priority due to higher thermal conductivity. The SRM made with the recipes 2, 4, 6, and 8 is the second priority due to lower thermal conductivity.

**Figure 6:**
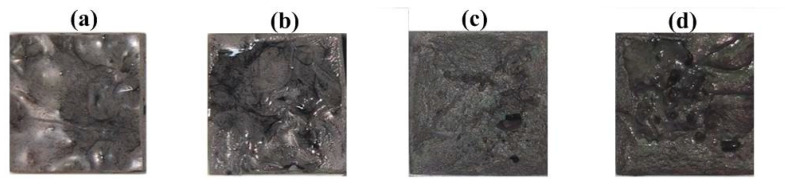
Top view of the specimens made with recipes (**a**) 3, (**b**) 5, (**c**) 7, and (**d**) 9.

**Figure 7:**
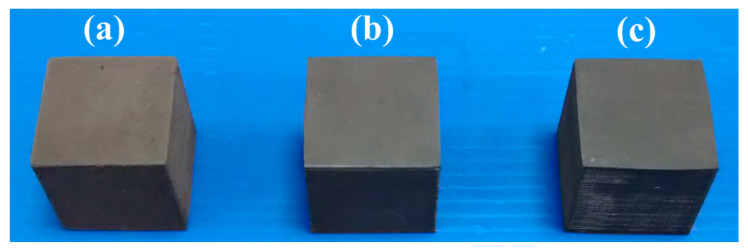
Specimens made with recipes (**a**) 6, (**b**) 4, and (**c**) 2.

Figure 6 shows the top view of the specimens made with recipes 3, 5, 7, and 9. The results showed that the specimens made with recipes 3, 5, 7, and 9 cannot be formed into cubic due to the poor fluidity of the mixture. Figure 7 shows the specimens made with recipes 6, 4, and 2. As can be seen, specimens can be formed into cubic successfully using recipes 6, 4, and 2. This means that recipes 3, 5, 7, and 9 can be used to fabricate high cooling efficiency SRM.

**Figure 8:**
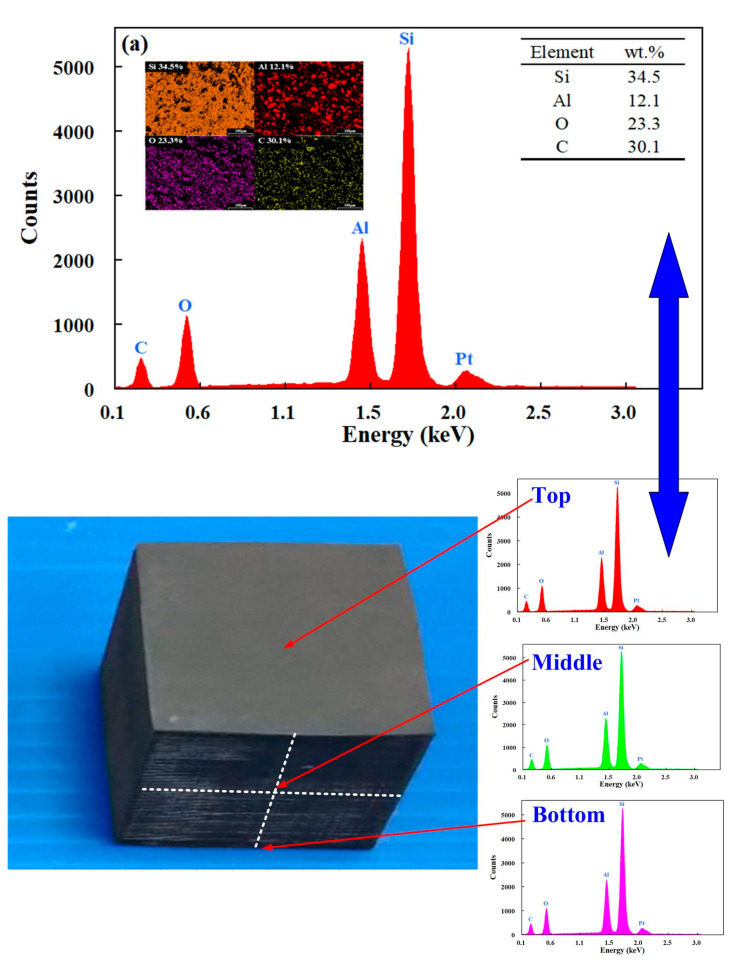

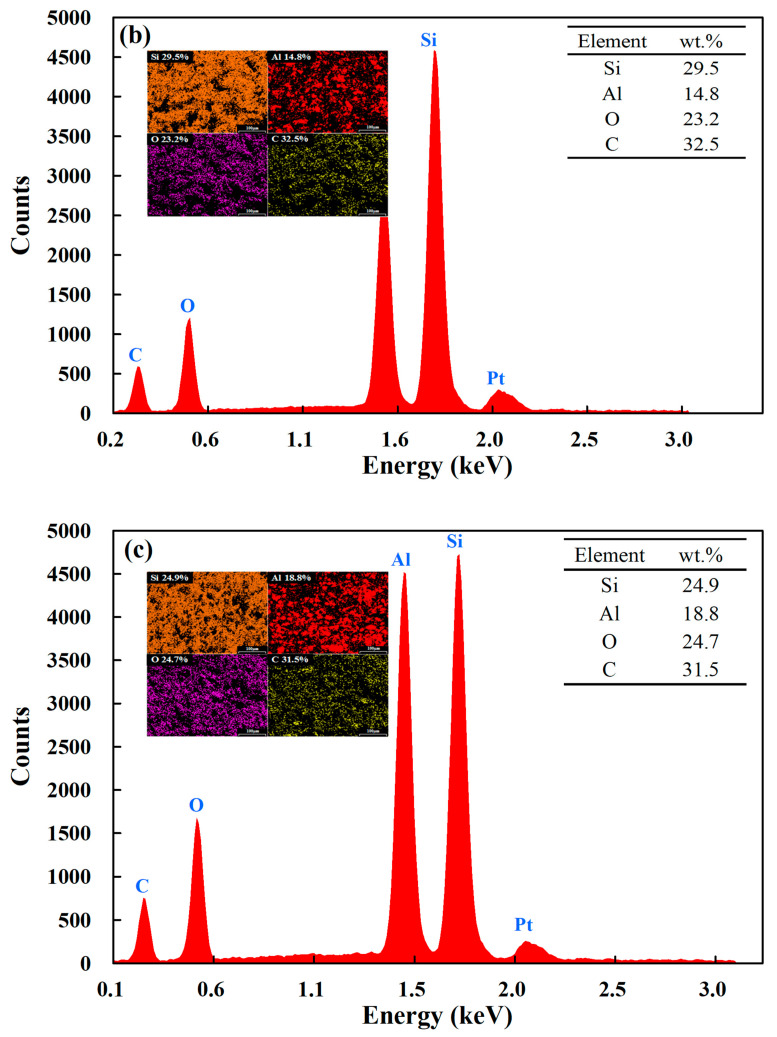
X-ray diffraction (XRD) patterns of the specimens made with recipes (**a**) 6, (**b**) 4, and (**c**) 2.

**Figure 9:**
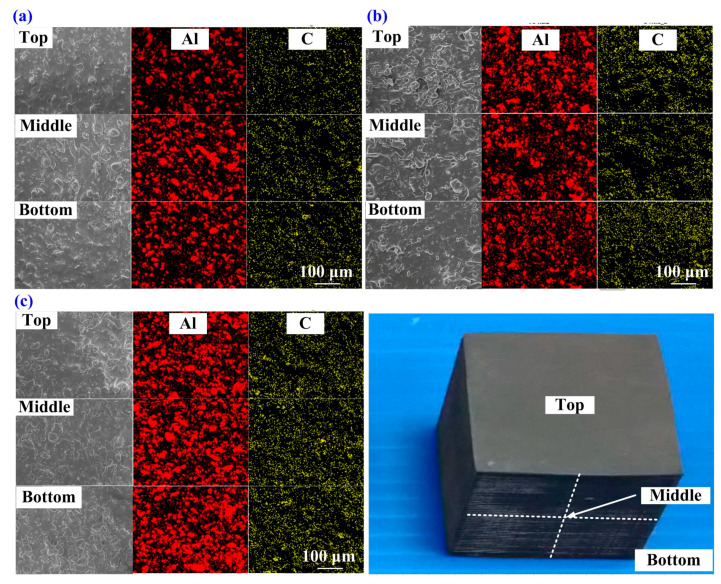
Energy-dispersive X-ray spectroscopy (EDS) images of the specimens made with recipes (**a**) 6, (**b**) 4, and (**c**) 2.

Figure 8 shows the XRD patterns of the specimens made with recipes 6, 4, and 2. The results clearly found that the stronger the signal strength of Al and G powders in the EDS spectrum stands for the more Al and G powders added to the SRM. To understand the dispersion of Al and G powders of the specimens made with recipes 6, 4, and 2, three positions, namely top, middle, and bottom were conducted by EDS. Figure 9 shows the EDS images of the specimens made with recipes 6, 4, and 2. As can be seen, the Al and G powders were distributed uniformly inside the specimens made with recipes 6, 4, and 2. This result indicates that no any agglomeration [37] and precipitation [38] of the Al and G powders inside the specimens were found.

**Figure 10:**
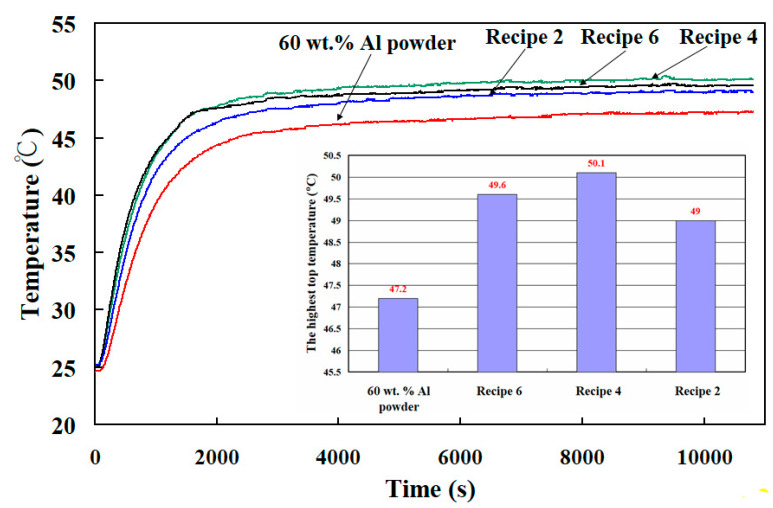
The top temperature of the specimens as a function of time.

**Figure 11:**
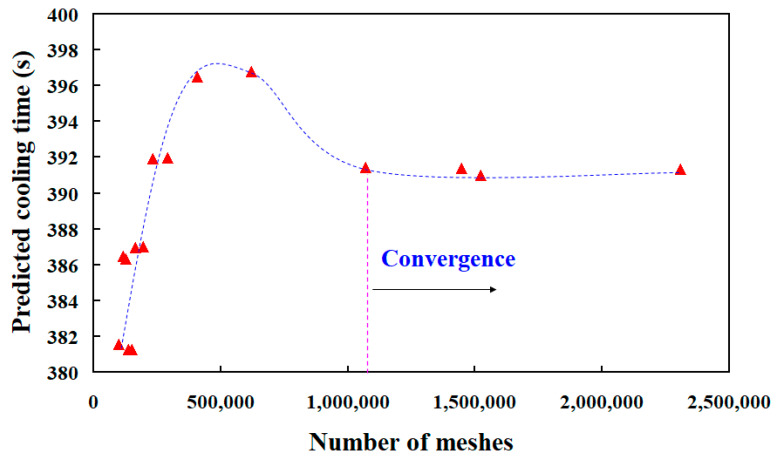
Number of meshes as a function of the cooling time of the injection molded product.

To understand the thermal conductivity of specimens, four different kinds of specimens were used to study. The heating temperature was fixed at 60 °C, and the test piece was heated and measured for 3 h. Figure 10 shows the top temperature of the specimens as a function of time. The highest top temperature of the specimen made with 60 wt.% Al powder, recipe 6, recipe 4, and recipe 2 are about 47.2 °C, 49.6 °C, 50.1 °C, and 49.0 °C, respectively. The thermal conductivities of the specimen made with 60 wt.% Al powder, recipe 6, recipe 4, and, recipe 2 are about 29.4 W/m-k, 34.2 W/m-k, 38.0 W/m-k, and 36.2 W/m-k, respectively. It was found that the thermal conductivity of the specimen made with recipe 4 was the highest. As can be seen, a significant improvement in the thermal conductivity about 77.6% can be obtained. The recipe 4 is composed of 52.6 wt.% Al powder, 5.3 wt.% G powder, and 42.1 wt.% liquid silicon rubber. Therefore, recipe 4 was used to fabricate the high cooling efficiency SRM. According to the thermal conductivity obtained, the simulation software entitled Moldex 3D was then employed to predict cooling time of the injection molded product, part temperature distribution, mold temperature distribution, and warpage of the wax patterns. In this study, the three-dimensional tetrahedron elements were selected because it has linear interpolation shape functions [39]. To investigate the optimal number of meshes, the mesh independent study was carried out with fifteen different numbers of meshes. Figure 11 shows the number of meshes as a function of the cooling time of the injection molded product. As can be seen, the cooling time of the injection molded product reached convergence when the mesh element counts exceed 1071.508. Thus, the mesh with 1071.508 cells was selected as the mesh structure based on both the correctness of the cooling time and the computing time.

**Figure 12:**
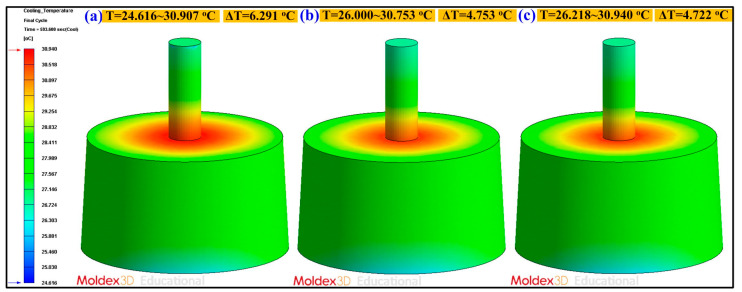
Numerical simulation results of the part temperature difference for (**a**) conventional silicone rubber mold (SRM), (**b**) SRM with 60 wt.% Al powder, (**c**) SRM made with recipe 4.

**Figure 13:**
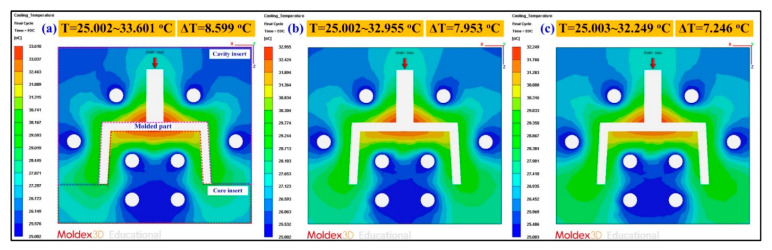
Numerical simulation results of the mold temperature difference for (**a**) conventional SRM, (**b**) SRM with 60 wt.% Al powder, (**c**) SRM made with recipe 4.

**Figure 14:**
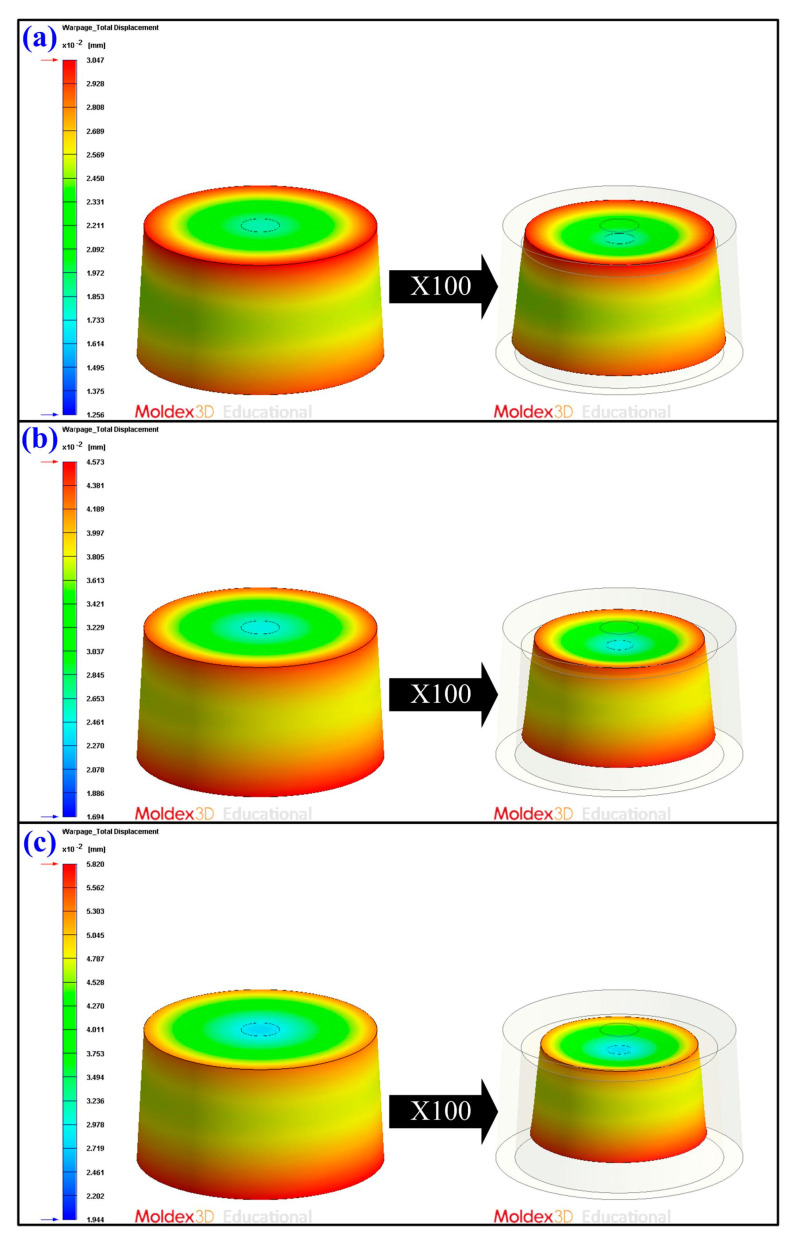
Numerical simulation results of the warpage of the molded parts for (**a**) conventional SRM, (**b**) SRM with 60 wt.% Al powder, (**c**) SRM made with recipe 4.

Figure 12 shows the numerical simulation results of the part temperature difference for conventional SRM, SRM with 60 wt.% Al powder, and SRM made with recipe 4. The part surface temperature distributions of conventional SRM, SRM with 60 wt.% Al powder, and SRM made with recipe 4 are 24.616–30.907 °C, 26.000–30.753 °C, and 26.218–30.940 °C, respectively. As can be seen, the part temperature differences for conventional SRM, SRM with 60 wt.% Al powder, and SRM made with recipe 4 are 6.291 °C, 4.753 °C, and 4.722 °C, respectively. This means that the part temperature difference for SRM made with recipe 4 has the lowest surface temperature difference of the molded part. Mold temperature difference stands for the temperature difference between the cavity plate and core plate. Generally, large mold temperature difference will result in part warpage. Figure 13 shows the numerical simulation results of the mold temperature difference for conventional SRM, SRM with 60 wt.% Al powder, and SRM made with recipe 4. The mold surface temperature distributions of conventional SRM, SRM with 60 wt.% Al powder, and SRM made with recipe 4 are 25.002–33.601 °C, 25.002–32.955 °C, and 25.003–32.249 °C, respectively. The mold temperature differences for conventional SRM, SRM with 60 wt.% Al powder, and SRM made with recipe 4 are 8.599 °C, 7.953 °C, and 7.246 °C, respectively. It is interesting to note that the mold surface temperature difference of the SRM made with recipe 4 is the lowest because the heat of the injected product can be absorbed and dissipated efficiently to the mold due to high thermal conductivity. This means that lower difference in the mold surface temperature difference will help improve the quality of the molded parts will be due to less warpage [40,41,42]. Figure 14 shows the numerical simulation results of the warpage of the molded parts for conventional SRM, SRM with 60 wt.% Al powder, and SRM made with recipe 4. The total displacements of the molded parts for conventional SRM, SRM with 60 wt.% Al powder, and SRM made with recipe 4 are 1.791 × 10^−2^ mm, 2.879 × 10^−2^ mm, and 3.876 × 10^−2^ mm, respectively. As can be seen, the SRM made with recipe 4 has larger warpage [43] since it was attributed to the higher volumetric shrinkage [44,45] caused by higher gate solidification speed [46,47] during the solidification stage of the molded part. This disadvantage can be solved by controlling packing pressure [48,49,50] appropriately during the packing stage for an injection mold with high cooling efficiency before demolding of the molded parts.

**Figure 15:**
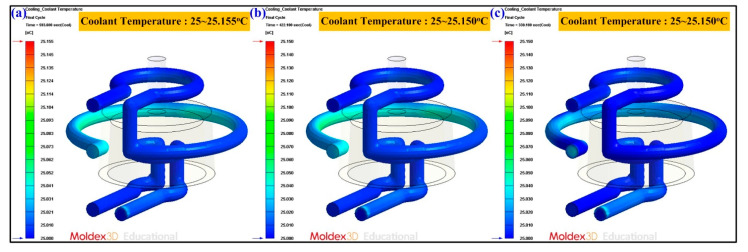
Numerical simulation results of the coolant temperature difference for (**a**) conventional SRM, (**b**) SRM with 60 wt.% Al powder, and (**c**) SRM made with recipe 4.

**Figure 16:**
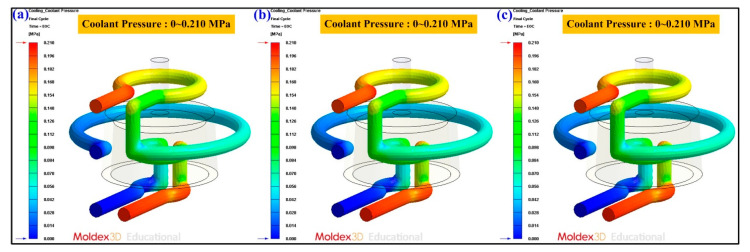
Numerical simulation results of the coolant pressure difference for (**a**) conventional SRM, (**b**) SRM with 60 wt.% Al powder, and (**c**) SRM made with recipe 4.

**Figure 17:**
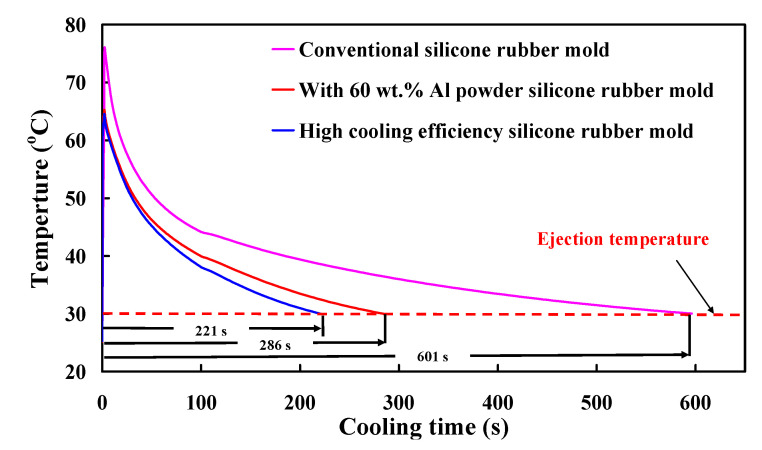
Numerical simulation results of the temperature of the molded wax pattern as a function of the cooling time.

**Figure 18:**
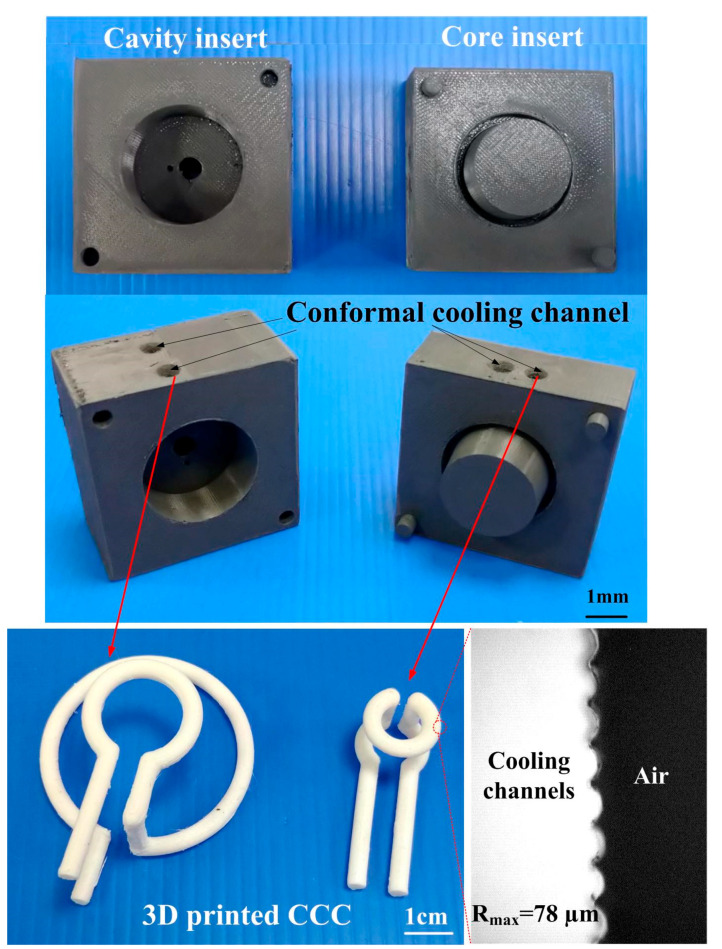
SRM made with recipe 4.

Cooling channel pressure result reveals the pressure distribution inside the cooling channels. In general, higher pressure in the coolant means higher energy in pumping to maintain the flow rate and cooling efficiency. The coolant temperature difference indicates the difference between the highest coolant temperature and the lowest coolant temperature. Figure 15 shows the numerical simulation results of the coolant temperature difference for conventional SRM, SRM with 60 wt.% Al powder, and SRM made with recipe 4. The coolant temperature difference was about 0.15 °C. As can be seen, there is no significant difference in the coolant temperature difference between three molds. Figure 16 shows the numerical simulation results of the coolant pressure difference for conventional SRM, SRM with 60 wt.% Al powder, and SRM made with recipe 4. The coolant pressure difference was about 0.21 MPa. As can be seen, there is no significant difference in the coolant pressure difference between three molds. According to the results described above, this means the layout of the CCC is appropriate. Figure 17 shows the numerical simulation results of the temperature of the molded wax pattern as a function of the cooling time. The cooling time of the injection molded product for conventional SRM, SRM with 60 wt.% Al powder, and SRM made with recipe 4 are around 601 s, 286 s, and 221 s, respectively. It was found that SRM made with recipe 4 can save the cooling time of the injection molded product about 380 s compared with the conventional SRM. Figure 18 shows the SRM made with recipe 4.

**Figure 19:**
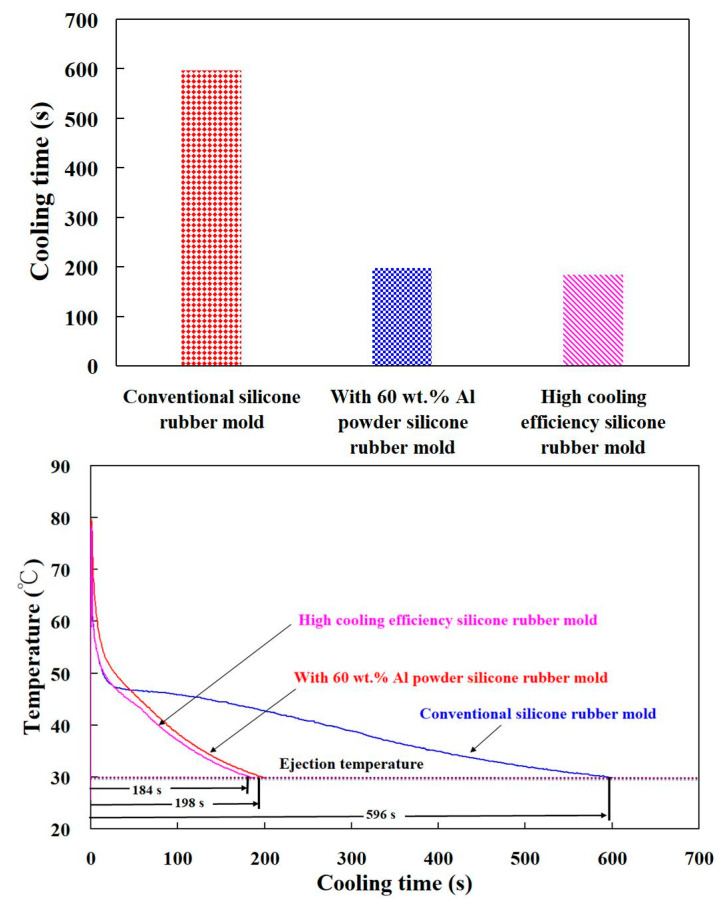
Temperature of the molded wax pattern as a function of actual cooling time.

**Figure 20:**
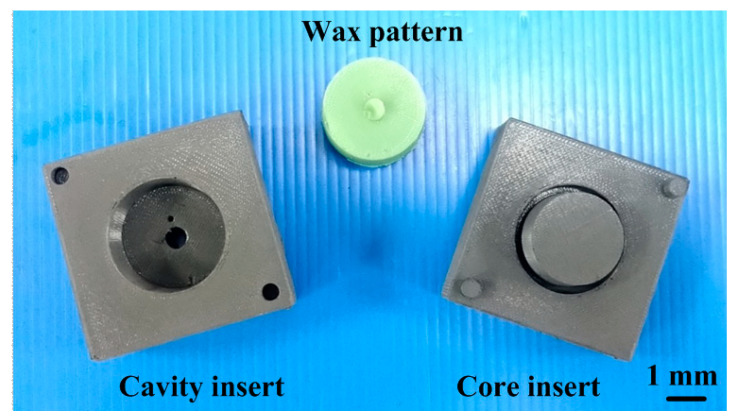
Photo of a molded wax pattern.

**Figure 21:**
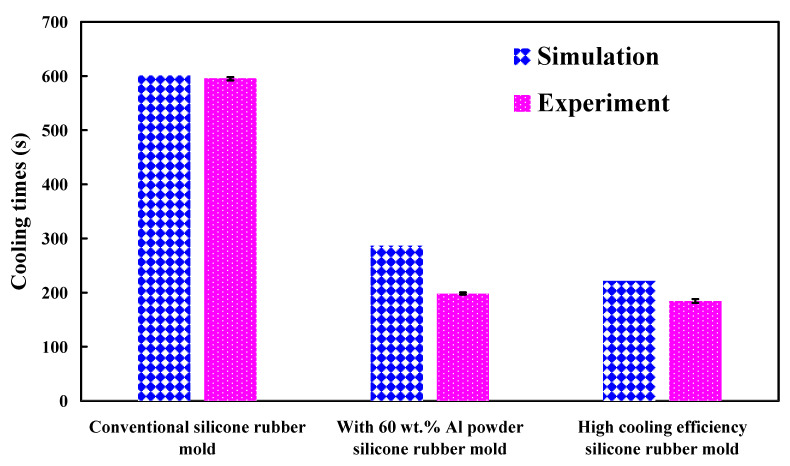
Comparison of the predicted and actual cooling time of injection molded products.

Figure 19 shows the temperature of the molded wax pattern as a function of actual cooling time. The cooling time of the injection molded product for conventional SRM, SRM with 60 wt.% Al powder, and SRM made with optimal formulation are around 596 s, 198 s, and 184 s, respectively. The results revealed that SRM made with optimal formulation can save the cooling time of the injection molded product up to 69.1% compared to conventional SRM. In addition, SRM made with optimal formulation can save the cooling time of the injection molded product up to 66.8% compared to SRM with 60 wt.% Al. Two advantages were found from this result. First, it is interesting to note that the cooling time of the molded wax pattern fabricated by SRM made with the optimal formulation can be further saved by around 15.3% compared to that of the molded wax pattern fabricated by SRM with 80 wt.% Fe powders [36]. Figure 20 shows the photo of a molded wax pattern. Second, the rust was not observed in the wall of the cooling channels of SRM after injection molding compared to SRM with 80 wt.% Fe powders [36]. Figure 21 shows the comparison of the predicted and actual cooling time of injection molded products. Two phenomena were found. It was found that the cooling time obtained by the Moldex3D simulation software is greater than that obtained by the experiment. The relative error rates of the cooling time between the experiment and numerical simulation were about 1%, 45%, and 20%, respectively. This can be attributed to the difference between the experimental conditions and characteristic parameters such as molding material, mold materials, and responses of the injection machine used in the simulation software [51,52,53].

**Figure 22:**
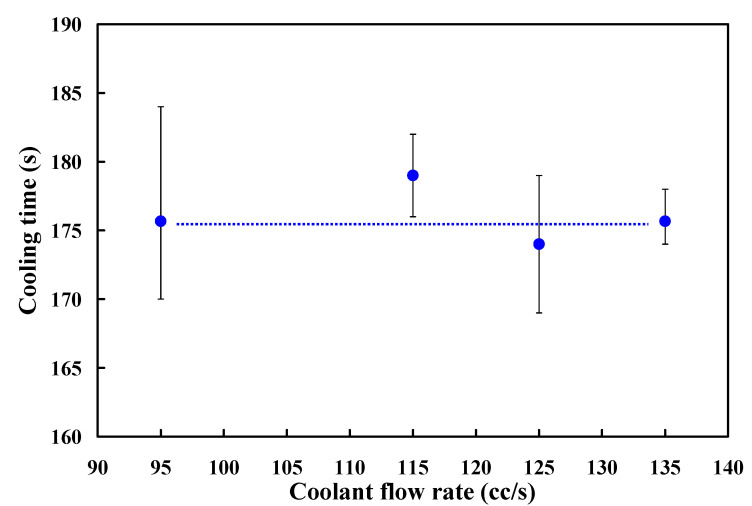
Cooling times of the molded parts as a function of different coolant flow rates.

Cooling channel flow rate means the volumetric flow rate along the cooling channel. In general, higher flow rate will result in higher heat transfer. In practice, the cooling time plays an important role in the cycle time. Generally, the turbulent flow provides approximately three to five times as much heat transfer as laminar flow. To understand the effects of different coolant flow rates on the cooling time of the molded parts for high cooling efficiency SRM, four different coolant flow rates were carried out in this study. The Reynolds numbers for coolant flow rates of 95 cc/s, 115 cc/s, 125 cc/s, and 135 cc/s are about 30,239, 36,606, 39,789, and 42,972, respectively. Figure 22 shows the cooling time of the molded parts as a function of different coolant flow rates. The average cooling time of the molded parts for the coolant flow rates of 95 cc/s, 115 cc/s, 125 cc/s, and 135 cc/s are 176 s, 179 s, 174 s, and 176 s, respectively. This means that changing the coolant flow rates has no significant effect on the cooling time of the molded parts because these four coolants have reached turbulent flow.

**Figure 23:**
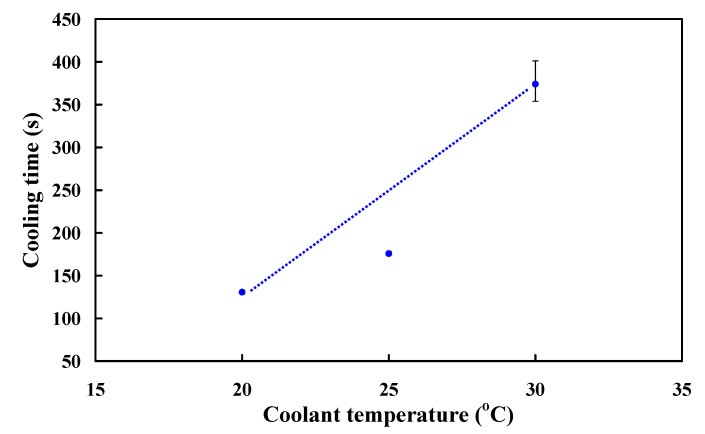
Cooling time of the molded parts as a function of different coolant temperatures.

The coolant temperature is an important topic on the cooling efficiency for SRM with CCC. Coolant temperature reveals the temperature distribution inside the cooling channels. With the same flow strength, in general, lower coolant temperature can result in higher cooling efficiency. To understand the effects of different coolant temperatures on the cooling time of the molded parts for high cooling efficiency SRM, three different coolant temperatures were carried out in this study. Figure 23 shows the cooling time of the molded parts as a function of different coolant temperatures. The average cooling time of the molded parts for the coolant temperatures of 20 °C, 25 °C, and 30 °C are 131 s, 176 s, and 374 s, respectively. As can be seen, the cooling time of the molded parts decreases with decreasing coolant temperature. This means that changing the coolant temperatures has a significant effect on the cooling time of the molded parts.

**Figure 24:**
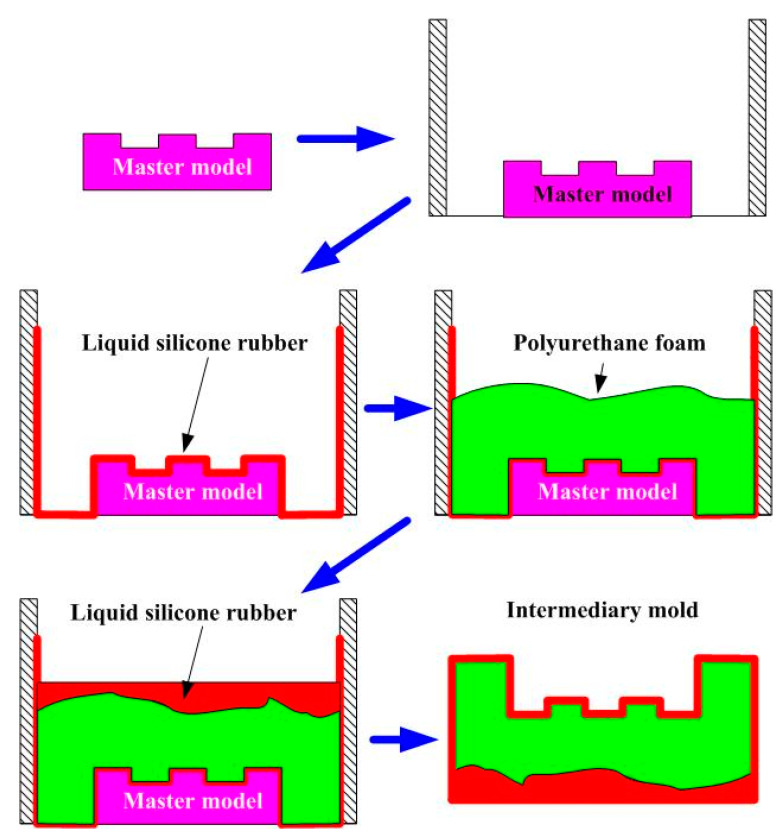
An innovative method for fabricating an intermediary mold for large rapid tooling (RT) with.

In general, an intermediary mold which is complementary in shape to the core or cavity inserts was commonly used for fabricating RT. However, the production costs of a large intermediary mold are costly. To reduce the production costs, this study demonstrated an innovative method using polyurethane foam as backing material to make intermediary mold. Figure 24 shows an innovative method for fabricating an intermediary mold for large RT. The use of polyurethane foam (PUF) in the manufacturing process of an intermediary mold can reduce the usage amount of new liquid silicone rubber significantly. An intermediary mold was then fabricated by liquid silicone rubber (LSR) and PUF. The LSR was first used as surface material of the intermediary mold and the polyurethane foam was then used as the backing material. Finally, the backside of the intermediary mold was sealed with LSR. This innovative method has application potential for the RT industry because of the production cost reduction increases with increasing the sizes of the intermediary mold.

The price–performance ratio is defined as the ratio of the reduction in cooling time to the production cost. The production costs for conventional SRM and SRM made with recipe 4 are NTD 12.5 and NTD 19.3, respectively. This means that the price–performance ratio of this SRM made by the optimal recipe is around 55. It should be noted that the SRM made with recipe 4 seems to be a promising method to manufacture wax patterns for manufacturing customized metal components through investment casting technology [54,55,56,57]. However, adding fillers into the SRM changes the material composition, which brings other problems, including wasted material recycling and environmental friendliness are also important research issues. In addition, the CCCs inside the SRM were not optimized. Thus, further works are required to optimize the layout of the CCCs using the simulation software. The mechanical properties of SRM are inferior to that fabricated by aluminum (Al)-filled epoxy resins [58,59,60,61]. Therefore, the Al-filled epoxy resins can be used to make an injection molding tool with CCC for batch production of wax patterns. Unfortunately, the CCC in the injection molding tool was not optimized. Thus, optimization of CCC is also an important research issue using the Taguchi method [62,63,64,65]. Further improvements in the mechanical properties of the SRM with CCC by adding fillers or reinforcing additives, such as wollastonite, glass fibers, carbon fibers, glass sphere, molybdenum disulfide [66,67,68,69], zirconia [70,71,72], silica sand, or silicon nitride [73,74,75] into the SRB is also an important research issue. In addition, the internal surface of CCC has high surface roughness [76,77,78] and result in reduction in mold life due to stress concentration [79,80,81,82]. Thus, surface improvement of the CCC by abrasive flow machining [83], electrochemical polishing [84], chemical polishing [85], laser polishing [86], ultrasonic cavitation abrasive finishing [87], or abrasive blasting [88] is also an important research topic. These issues are currently being investigated and the results will be presented in a later study.

## 4. Conclusions

The productivity was influenced by the cooling time significantly in the wax injection molding process since the cooling time accounts for approximately two thirds of the cycle time. Thus, improving the cooling efficiency of the SRM is an important research issue. The objective of this study is to develop a recipe for manufacturing low-cost SRM with high cooling performance and resistance to rust. The remarkable findings of this study can be used for the fabrication of SRM for trial production of wax patterns in the investment casting industry. Based on the results obtained in this study, the following conclusions can be drawn:

1. Improving cooling performance of injection molding tool with CCC by adding hybrid fillers has been demonstrated. An optimal recipe comprising 52.6 wt.% Al powder, 5.3 wt.% G powder, and 42.1 wt.% liquid silicon rubber can be used to make SRM with excellent cooling efficiency. The price–performance ratio of this SRM made by the proposed recipe is around 55.
2. The thermal conductivity of the SRM made by the proposed recipe can be increased by up to 77.6% compared with conventional SRM. In addition, the actual cooling time of the injection molded product can be shortened up to 69.1% compared with the conventional SRM.
3. For the SRM made by the proposed recipe, the relative error rate of the cooling time between the experiment and numerical simulation was approximately 20%.
4. Changing the coolant temperatures has a significant effect on the cooling time of the molded parts. However, changing the coolant flow rates has no significant effect on the cooling time of the molded parts.

## Figures and Tables

**Table 1:** Planning table for silicon rubber adding different weight ratios of Al powder and G powder.

Recipe	Al Powder	G Powder	SR	wt.%		
	g	g	g	Al	G	Silicone Rubber
1	60	0	40	60.0	0	40.0
2	60	5	40	57.1	4.8	38.1
3	60	10	40	54.5	9.1	36.4
4	50	5	40	52.6	5.3	42.1
5	50	10	40	50.0	10.0	40.0
6	40	5	40	47.1	5.9	47.1
7	40	10	40	44.4	11.1	44.4
8	30	5	40	40.0	6.7	53.3
9	30	10	40	37.5	12.5	50.0

## Data Availability

The data presented in this study are available on request from the corresponding author.
